# Systemic pro-inflammatory response facilitates the development of cerebral edema during short hypoxia

**DOI:** 10.1186/s12974-016-0528-4

**Published:** 2016-03-11

**Authors:** Ting-Ting Song, Yan-Hua Bi, Yu-Qi Gao, Rui Huang, Ke Hao, Gang Xu, Jia-Wei Tang, Zhi-Qiang Ma, Fan-Ping Kong, John H. Coote, Xue-Qun Chen, Ji-Zeng Du

**Affiliations:** Division of Neurobiology and Physiology, Department of Basic Medical Sciences, Institute of Neuroscience, School of Medicine, Key Laboratory of Medical Neurobiology of The Ministry of Health, Zhejiang Province Key Laboratory of Neurobiology, Zhejiang University, Hangzhou, 310058 China; Department of Pathophysiology and High Altitude Physiology, College of High Altitude Military Medicine, Third Military Medical University, Chongqing, 400038 China; School of Clinical and Experimental Medicine, University of Birmingham, Birmingham, B15 2TT UK

## Abstract

**Background:**

High-altitude cerebral edema (HACE) is the severe type of acute mountain sickness (AMS) and life threatening. A subclinical inflammation has been speculated, but the exact mechanisms underlying the HACE are not fully understood.

**Methods:**

Human volunteers ascended to high altitude (3860 m, 2 days), and rats were exposed to hypoxia in a hypobaric chamber (5000 m, 2 days). Human acute mountain sickness was evaluated by the Lake Louise Score (LLS), and plasma corticotrophin-releasing hormone (CRH) and cytokines TNF-α, IL-1β, and IL-6 were measured in rats and humans. Subsequently, rats were pre-treated with lipopolysaccharide (LPS, intraperitoneal (ip) 4 mg/kg, 11 h) to induce inflammation prior to 1 h hypoxia (7000 m elevation). TNF-α, IL-1β, IL-6, nitric oxide (NO), CRH, and aquaporin-4 (AQP4) and their gene expression, Evans blue, Na^+^-K^+^-ATPase activity, p65 translocation, and cell swelling were measured in brain by ELISA, Western blotting, Q-PCR, RT-PCR, immunohistochemistry, and transmission electron micrography. MAPKs, NF-κB pathway, and water permeability of primary astrocytes were demonstrated. All measurements were performed with or without LPS challenge. The release of NO, TNF-α, and IL-6 in cultured primary microglia by CRH stimulation with or without PDTC (NF-κB inhibitor) or CP154,526 (CRHR1 antagonist) were measured.

**Results:**

Hypobaric hypoxia enhanced plasma TNF-α, IL-1β, and IL-6 and CRH levels in human and rats, which positively correlated with AMS. A single LPS injection (ip, 4 mg/kg, 12 h) into rats increased TNF-α and IL-1β levels in the serum and cortex, and AQP4 and AQP4 mRNA expression in cortex and astrocytes, and astrocyte water permeability but did not cause brain edema. However, LPS treatment 11 h prior to 1 h hypoxia (elevation, 7000 m) challenge caused cerebral edema, which was associated with activation of NF-κB and MAPKs, hypoxia-reduced Na^+^-K^+^-ATPase activity and blood-brain barrier (BBB) disruption. Both LPS and CRH stimulated TNF-α, IL-6, and NO release in cultured rat microglia via NF-κB and cAMP/PKA.

**Conclusions:**

Preexisting systemic inflammation plus a short severe hypoxia elicits cerebral edema through upregulated AQP4 and water permeability by TLR4 and CRH/CRHR1 signaling. This study revealed that both infection and hypoxia can cause inflammatory response in the brain. Systemic inflammation can facilitate onset of hypoxic cerebral edema through interaction of astrocyte and microglia by activation of TLR4 and CRH/CRHR1 signaling. Anti-inflammatory agents and CRHR1 antagonist may be useful for prevention and treatment of AMS and HACE.

**Electronic supplementary material:**

The online version of this article (doi:10.1186/s12974-016-0528-4) contains supplementary material, which is available to authorized users.

## Background

Traveling to high altitude has become very fashionable in recent years, and ascending too fast or too high may cause the development of acute mountain sickness (AMS) due to hypobaric hypoxia. AMS is characterized by headache and usually accompanied by anorexia, nausea, sleep disturbance, malaise, or a combination of these symptoms. These relatively benign cerebral symptoms if not treated or treated inappropriately may develop into the more lethal high-altitude cerebral edema (HACE) [1–5]. HACE is characterized by nonspecific pathophysiological symptoms such as severe headache, loss of coordination, disturbances of consciousness, psychiatric changes, and coma [5, 6]. HACE occurrence is not fully predictable because the underlying molecular-cellular mechanisms contributing to these changes caused by exposure to severe high-altitude hypoxia are poorly understood. Under normobaric conditions, studies of the physiology and pathophysiology of the blood-brain barrier (BBB) show that brain edema occurs consequent to astrocyte swelling and increase in BBB permeability [7]. In hypobaric hypoxia, these latter pathological effects depend on upregulation of aquaporin-4 (AQP4) in astrocytes via activation of corticotrophin-releasing hormone receptor type 1 (CRHR1) following increased local secretion of corticotrophin-releasing hormone (CRH) in the brain [8]. This accords with several studies showing a critical role of upregulated-AQP4 in the formation of brain edema in various pathological clinical conditions including ischemia and trauma [9, 10]. Why and how these changes may progress at high altitude to more severe AMS and HACE is unclear.

HACE usually occurs in unacclimatized individuals at altitudes over 3000 m and even in seemingly well-acclimatized mountaineers at extreme altitudes of 7000 m [1]. The sudden onset of HACE remains a puzzle, and several studies have failed to identify the reasons. For example, a well-designed series of studies on humans exposed to hypobaric hypoxia (4875 m) measured molecular markers of oxidative stress and brain tissue damage [11, 12]. These studies indicated there is an increased vascular permeability and inflammatory response associated with mild brain swelling but there is no clear association with oxygen-derived free radicals. Other factors have been considered such as genetic predisposition, variation in hypoxia inducible factor activation, renin-angiotensin activation, and nitric oxide synthesis, but none are convincingly associated with pathophysiology of cerebral edema [6]. Thus, the mechanisms remain elusive, and effective strategies for prevention or therapy of HACE are presently limited. However, it has been suggested that infection-elicited inflammation is a trigger for HACE [3, 4, 13].

Previously, few studies have given much attention to the stress-related central nervous actions of CRH, which are also activated by systemic inflammation. Interestingly, CRH is locally released in the brain and specific tissues, and its receptor CRHR1 is known to be involved in enhancement of the immune and the inflammatory response [14, 15]. This is in contrast to the systemic indirect immunosuppressive action of the hormones [16, 17]. We have reported that CRH/CRHR1 overactivation by hypoxia induces cerebral edema and apoptosis [8]. However, whether an infective systemic inflammatory response interacting with an event induced by hypobaric hypoxia in the brain through CRH-stimulated activation of microglia to facilitate or to initiate HACE or not remains unknown.

In the present study, we address this question by first measuring pro-inflammatory factors and CRH in plasma of humans and rats to establish the changes that occur when they are exposed to high altitude. We then set up a rat model showing an infection-elicited systemic inflammation by LPS treatment following hypobaric hypoxia challenge results in onset of HACE, while each alone fails. We then addressed the underlying mechanisms. The results reveal mechanisms by which hypoxia-triggered CRH-CRHR1 signaling activates pathways in brain microglia and show how this is enhanced by inflammation leading to the development of cerebral edema which if severe enough could be responsible for HACE in humans.

## Methods

### Human hypoxia exposure

The human’s study was approved by the Ethics Committee of the Third Military Medical University (LKSD2014001). Seventy-four healthy volunteers were recruited (male, 18–23 years old) in Chengdu, China, at 540 m altitude, as lowland controls, and a written informed consent was received by all of the volunteers prior to inclusion in the study. Fasting blood samples from all volunteers were collected in the morning (07:00–08:00) in Chengdu and the third morning at Rikaze (3860 m altitude), Tibet Autonomous Region, China. The Lake Louise Score (LLS) [18] for each participant was recorded by a clinician. Plasma was obtained by centrifugation and stored at −80 °C.

### Animals

Male Sprague-Dawley rats weighing 210 ± 10 g were supplied by the Shanghai Experimental Animal Center, Chinese Academy of Sciences, Shanghai, China (SCXK2012-0002). Animals were maintained under constant conditions (temperature 20 ± 2 °C, humidity 40–60 %, light/dark cycle 12 h) and fed with a standard diet and water. Rats were housed in the lab for 2 weeks to adapt them to the environment before hypoxic experiment. All experiments were conducted in accordance with the NIH laboratory animal care guidelines. All protocols concerning animal use were approved by the Zhejiang University Animal Care and Use Committee (ZJU201304-1-01-025).

### Animal hypoxia treatment

Rats in the hypoxia group were placed in a hypobaric chamber (Avic Guizhou Fenglei Aviation Armament Co., Ltd, China, FLYDWC-50-IIC) and exposed to hypobaric hypoxia of 7000 m altitude (41.02 kPa, 8.2 % O_2_) for 1, 4 or 8 h with or without CP154,526 (CRHR1 antagonist, 30 mg/kg, intraperitoneal (ip)) or 5000 m altitude (54.02 kPa, 10.8 % O_2_) for 2 days [19]. The normoxia group was placed in the same chamber set at sea level (100.08 kPa, 20.9 % O_2_).

Rats were randomized into six groups. (1) The control group (*n* = 12) was injected with 0.9 % saline. (2) The LPS group (*n* = 6) was injected with LPS (ip, 4 mg/kg, body weight) [20–22] for 12 h. (3) The hypoxia group (*n* = 6) was exposed to hypoxia (altitude of 7000 m) for 1 h (injected with 0.9 % saline). (4) The LPS + hypoxia group (*n* = 12) was injected with LPS for 11 h then exposed to hypoxia for 1 h. (5) The pyrrolidine dithiocarbamate (PDTC, NF-κB inhibitor) + LPS + hypoxia group (*n* = 10) was pre-treated with PDTC for 0.5 h, then followed by LPS treatment for 11 h, again followed by hypoxia for 1 h. (6) The PDTC + hypoxia group (*n* = 6) was injected with PDTC for 0.5 h then exposed to hypoxia for 1 h.

### Reagents, antibodies and plasmid

LPS was purchased from Sigma (*Escherichia coli* 055:B5, USA). IL-1β and TNF-α were from Peprotech (USA). Evans blue, the NF-κB inhibitor PDTC, the p38 inhibitor SB203580, the ERK1/2 inhibitor U0126, and the p300 inhibitor curcumin were from Sigma (USA). The JNK inhibitor SP600125 was from Cell Signaling Technology (USA). A fluorescent dye calcein-AM used for live cell imaging was from Invitrogen (USA). The antibodies against p65, p38, phospho-p38, p44/42 MAPK, phospho-p44/42 MAPK, JNK, phospho-JNK, GFAP, and NF-κB were from Cell Signaling Technology (USA). The AQP4 antibodies were from Chemicon (USA). CRH was obtained from Tocris (1151, UK), and CRHR1 antagonist, CP154,526, was kindly donated by the Pfizer company (USA). The complementary DNA (cDNA) sequences encoding rat AQP4 (NM_001142366) were synthesized (Sangon Biotech, Shanghai, China) and then inserted into pmRFP-C1 at the XhoI and EcoRI sites.

### Pharmaceutical treatment

For in vivo studies, LPS and PDTC were dissolved in 0.9 % saline. Rats received ip injection of LPS (4 mg/kg), PDTC (150 mg/kg), CP154,526 (ip, 30 mg/kg), or vehicle. For in vitro studies, astrocytes were treated with LPS (0.5 μg/ml), IL-1β (0.1 μg/ml) or TNF-α (0.1 μg/ml), and PDTC (100 μM) for 0.5 h. Microglia were incubated with CRH (10^−15^ M) for 12 h and CRHR1 (100 nM) antagonist for 0.5 h.

### Electron microscopy

Preparation of samples for transmission electron microscopy was performed as previously described. Rats were anesthesized and perfused first by PBS, followed by fixation using 2.5 % (*w*/*v*) glutaraldehyde. Samples were fixed in 2.5 % glutaraldehyde and then postfixed in 1 % buffered osmium tetroxide, dehydrated in ethanol, and embedded in epoxy resin. Ultrathin sections (80 nm) were stained with 2 % uranyl acetate and 3 % lead citrate and examined in a TECNAI 10 transmission electron microscope.

### siRNA for p300

siRNA duplexes were from GenePharma (Shanghai GenePharma Co., Ltd, China). Transient transfection of siRNA or RFP-AQP4 was performed with Lipofectamine 2000 according to the manufacturer’s instructions (Invitrogen, USA). Astrocytes were analyzed at 48 h after transfection of siRNA, and for HeLa cells at 24 h after transfection of RFP-AQP4. The following siRNA duplexes were used: GCCCUGGCAAUAUAUAGAUTT for p300 and UUCUCCGAACGUGUCACGUTT for negative control siRNA.

### Immunofluorescence staining and fluorescence microscopy

For brain cortex tissue, 10-μm frozen sections were cut by cryotome (HM505E, Germany). Immunofluorescence staining of the tissue sections and cells was performed as described previously. In brief, tissue sections and astrocytes were fixed in 4 % formaldehyde. After being washed three times in PBS, samples were incubated in PBS/FBS (PBS, pH 7.4, containing 10 % FBS) to block nonspecific sites of antibody adsorption. The samples were then incubated with appropriate primary antibodies (GFAP, 1:100, p65 1:100) and secondary antibodies (Alexa Fluor 488- and 545-tagged, 1:500) in 0.1 % saponin. Images were acquired on a scanning confocal microscope (LSM 510; Carl Zeiss, Germany) and analyzed with the LSM 510 software. The number of astrocytes with p65 translocation was scored by the immunofluorescence signal in the nucleusor cytosol.

### Western blotting and cytokine assay

Total protein was extracted from rat brain cortex or astrocytes. Sample aliquots of proteins were denatured and loaded on sodium dodecylsulfate-polyacrylamide gel. Proteins were then transferred to a polyvinylidene difluoride membrane. After being blocked in TBST containing 5 % bovine serum albumin or milk, the membrane was stained with the specific primary antibody followed by HRP-conjugated goat-anti-rabbit or goat-anti-mouse antibody. The specific bands were detected by an ECL Western blotting detection system (Amersham Bioscience, USA). Rat and human TNF-α, IL-1β, and IL-6 in serum and brain cortex tissue were tested by ELISA (RayBiotech, USA and Minneapolis, MN, USA) according to manufacturer’s instructions.

### NO assay

In 96-well plates, 50 μl of cell supernatant were added and reacted with an equal volume of Griess reagent (Beyotime, China) for 10 min at room temperature in the dark. Absorbance at 570 nm was determined using a microplate reader.

### Q-PCR and RT-PCR

Total RNA was isolated from cultures with the RNeasy mini kit (Qiagen, USA), as per the manufacturer’s protocol. RNA was quantified with a spectrophotometer (Nanodrop ND-1000, Thermo Scientific, USA). Reverse transcription of RNA to cDNA was achieved using 2 μg of total RNA with random hexamer primers using the TransScript Two-Step RT-PCR SuperMix Kit (TransGen Biotech, China). Only RNA with a 260/280 ratio of 1.9–2.0 was used for cDNA synthesis and PCR analysis. Q-PCR was performed with SYBR premix Ex TaqTM (Takara, China) on CFX96 Touch™ Real-Time PCR Detection System (Bio-Rad, USA). The primers used are presented as following:

- Rat *AQP4*: (F) TGGTCCTCATCTCCCTCTGCTT, (R) TGAACCGTGGTGACTCCCAATCC
- Rat *TNF-α*: (F) CCACGCTCTTCTGTCTACTG, (R)GCTACGGGCTTGTCACTC
- Rat *leave a blank space IL-1β*: (F) CTTCAAATCTCACAGCAGCATC, (R) GCTGTCTAATGGGAACATCACA
- Rat *IL-6*: (F) AGCCACTGCCTTCCCTAC, (R) TTGCCA TTGCACAACTCTT
- Rat *CRH*: (F) AAAATGTGGATCCAAGGAGGA, (R) TAGCCACCCCTCAAGAATGAA
- Rat 18s: (F) GTAACCCGTTGAACCCCATT, (R) CCATCCAATCGGTAGTAGCG

### Brain water content

After the end of experiments, rats were sacrificed by decapitation and the brain was immediately removed and weighed and then dried in a thermostatic oven for 48 h at 120 °C until drying to a constant weight. The dried brain was re-weighed and the percent brain water content (BWC) calculated as [(wet weight − dry weight) / wet weight] × 100 % [8].

### Blood-brain barrier permeability

BBB permeability was determined by Evans blue extravasations. Briefly, the dye (2 %, 2 ml/kg) was injected via the tail vein and circulated for 2 h. Rats were deeply anesthetized and perfused with saline to remove intravascular dye. The brains were removed and weighed. The dye in the brain was extracted by incubating in formamide (1 ml/100 mg) for 24 h at 65 °C, and the samples centrifuged. The supernatant was measured for absorbance of Evans blue at 630 nm, and dye concentrations were determined by a standard curve. The concentration of the dye (Evans blus) was expressed as micrograms of the dye per gram wet weight of the tissue.

### Cell culture and transfections

Astrocyte cultures were prepared from brains of 1-day-old neonatal Sprague-Dawley rat pups, and cultures were grown in DMEM containing penicillin, streptomycin, and 10 % fetal bovine serum, incubated at 37 °C in a humidified incubator provided with 5 % CO_2_ and 95 % air. After 12 days, cultures were shaken at 260 rpm for 12 h at 37 °C to purify the astrocytes. Cell populations consisted of over 98 % astrocytes as determined by immunocytochemical examination with anti-GFAP. HeLa cells and astrocytes were, respectively, cultured in DMEM supplemented with 10 % fetal bovine serum (FBS) at 37 °C under 5 % CO_2_.

Microglia cultures were prepared from the cortex of 1-day-old Sprague-Dawley rat pups [23, 24]. The isolated cells were plated in PDL-coated 75 cm^2^ culture flasks in minimum essential medium (MEM) containing 10 % FBS, 50 U/ml penicillin, and 50 μg/ml streptomycin. Cells were maintained in a humidified atmosphere of 5 % CO_2_, 95 % air at 37 °C. At 10 days, the microglia were separated from other cells by washing and shaking for 10 min (with medium changes at 24 h and then every 3 days). The detached cells were plated on PDL-coated cover slips in a 24-well plate at a density of 1 × 10^5^ per well. After 1 h incubation at 37 °C, the nonadherent cells were removed and the adherent cells were fed with DMEM containing 10 % FBS for 24 h before further study. The purity of microglia was consistently >98 %, as determined by flow cytometry quantification of CD11b^+^ cells.

### Measurement of membrane water permeability

Cells were mounted in a closed chamber on the stage of an inverted confocal laser scanning microscope (Nikon TE2000, Japan) in isosmotic, 300 mOsm Hanks, loaded with 20 μM calcein-AM (Molecular Probes, Invitrogen, USA) for 5 min at room temperature, and then washed with Hanks. The cells were perfused with isosmotic Hanks and scanned every 1.8 s with excitation at 488 nm. The fluorescent signal was collected at 515–525 nm from an optical slice within the cell body. Cells were then subjected to an osmotic shock by switching the perfusate to a hypo-osmotic, 200 mOsm Hanks. Cell swelling was monitored as a decrease of calcein fluorescence, which occurred due to the dilution of the fluorophore and a reduction in self-quenching. The initial slope of the fluorescence intensity curve was used to calculate water permeability (Pf) of each cell as described in detail previously [8, 25].

### Statistical analysis

In human data, paired two-tailed *t* test was used for the same group, unpaired two-tailed *t* test was used between AMS group and no-AMS group, and unpaired two-tailed *t* test was used in no-AMS followed by Bonferroni’s multiple comparisons test (*p* values less than 0.017 were regarded to reflect a significant difference). The correlations between plasma cytokines and LLS score were analyzed by using *Spearman* correlation. In rat data, comparisons between two groups were analyzed by an unpaired two-tailed Student’s test; in the case of multiple mean comparisons, the data were analyzed by one-way ANOVA or two-way ANOVA (GraphPad Prism 6). *p* values less than 0.05 were regarded to reflect a significant difference. Data are presented as means ± SD.

## Results

### Hypoxia induces inflammatory response and CRH release in humans and rats and correlates with human AMS

**Fig. 1:**
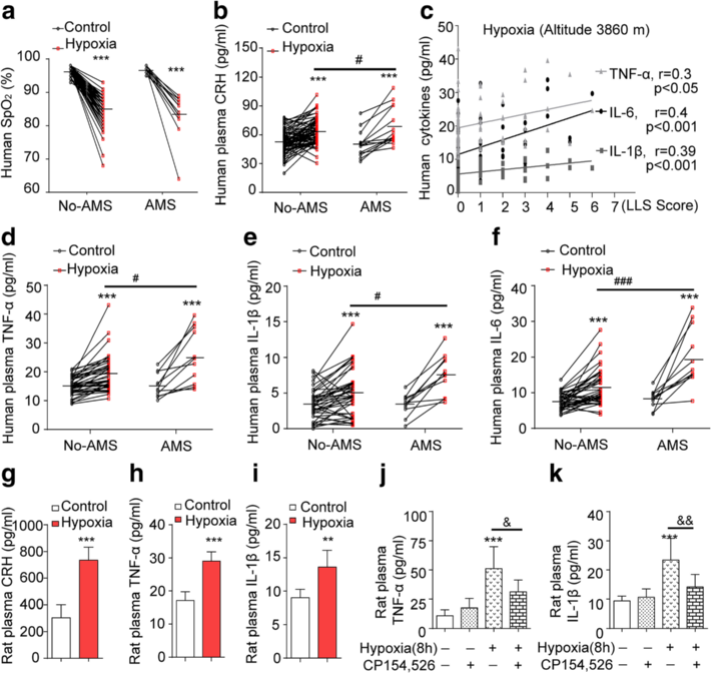
High-altitude hypoxia induced increased levels of TNF-α, IL-1β, IL-6, and CRH in humans and rats. **a** Human SpO_2_ was measured by fingertip pulse oximeter at low and high altitudes. **b** The levels of CRH in plasma of all human volunteers was determined by ELISA (*n* = 74). **c** The correlation between LLS score data and cytokines (TNF-α, IL-1β, and IL-6) level at high altitude by *Spearman* correlation analysis. **d**–**f** The levels of TNF-α, IL-1β, and IL-6 in plasma of all human volunteers was determined by ELISA (*n* = 74). ***P < 0.001 compared with control (low altitude, 540 m); ^#^ *P* < 0.05, ^###^ *P* < 0.001 compared with no-AMS group at high altitude (3860 m). **g**–**i** The rats in hypoxia group were exposed to hypoxia at altitude of 5000 min a hypobaric chamber for 2 days. The levels of CRH, TNF-α, and IL-1β in plasma were measured (*n* = 7). **j**, **k** Hypoxia-increased TNF-α and IL-1β levels in plasma were blocked by CRHR1 antagonist CP154,526 (*n* = 7). **P < 0.01; ***P < 0.001 compared with control, ^&^ *P* < 0.05 (hypoxia vs hypoxia + CRHR1 antagonist); the data are presented as mean ± SD

Fasting blood samples from all volunteers (*n* = 74) were collected at a lowland location and highland location on the Tibetan plateau. The incidence of AMS was 44.6 % at day 1, 21.6 %, at day 2, and 14.9 % at day 3, which gradually reduced with extension of arriving at altitude of 3860 m (Table 1). At the altitude of 3860 m, blood SpO_2_ levels were reduced in all volunteers, but there was no difference between no-AMS and AMS group when measured at day 3 (Fig. 1a). Plasma CRH levels were increased in volunteers with AMS or no-AMS but were significantly higher in those with AMS at day 3 (Fig. 1b). The correlation between LLS and cytokine level at day 3 by using *Spearman* correlation analysis showed there was a positive correlation between LLS and cytokine levels of TNF-α, IL-1β, and IL-6 at high altitude (*r* = 0.3, *p* < 0.05; *r* = 0.4, *p* < 0.001; *r* = 0.39, *p* < 0.001, respectively, Fig. 1c) but no correlation at low land. TNF-α, IL-1β, and IL-6 levels in the plasma of all volunteers were significantly increased at altitude of 3860 m at day 3 (^#^*P* < 0.05, ^###^*P* < 0.001, respectively, Fig. 1d–f), and the increases were significantly higher in volunteers with AMS than no-AMS. At altitude of 3860 m, at day 3, plasma TNF-α, IL-1β, and IL-6 levels were significantly higher in the AMS groups than in the no-symptoms group (A) within the no-AMS groups (Table 2, E vs A). In addition, the IL-6 levels in the headache-only group (B) were significantly higher than those in the no-symptom group (A) (^+^*p* = 0.008, B vs A). Headache + two symptoms group (E) of AMS groups was higher than in the other symptoms group (D) (^&^*p* = 0.02, D vs E); moreover, IL-1β levels were higher (increased trend) in headache + one symptom group (C) and other symptoms group (D) than in the no-symptom group (A) (^$^*p* = 0.013 and ^@^*p* = 0.03; C vs A and D vs A), suggesting that high-altitude hypoxia-increased plasma levels of pro-inflammatory factors (TNF-α, IL-1β, and IL-6) are associated with AMS. Interestingly, exposure of rats to hypobaric hypoxia (10.8 % O_2_, at altitude of ~5000 m) for 2 days increased plasma levels of CRH, TNF-α, IL-1β (Fig. 1g–i), and hypoxia (7000 m, 8 h)-increased plasma levels of TNF-α and IL-1β were blocked by CRHR1 antagonist (CP154,526) (Fig. 1j, k), suggesting that hypobaric hypoxia induces an leave a blank spaceimmune-inflammatory response in humans and rats, which is positively associated with activation of CRH/CRHR1 and with the development of AMS in humans.

**Table 1:** Comparison of AMS and no-AMS (total *n* = 74) volunteers at an altitude of 3860 m

	day 1	day 2	day 3
AMS	33/74 (44.6 %)	16/74 (21.6 %)	11/74 (14.9 %)
Headache	33/33 (100 %)	16/16 (100 %)	11/11 (100 %)
No-AMS	41/74 (55.4 %)	58/74 (78.4 %)	63/74 (85.1 %)
Headache only	8/41 (19.5 %)	7/58 (12.0 %)	6/63 (14.6 %)
Headache + one symptom	9/41 (21.9 %)	9/58 (15.5 %)	3/63 (4.8 %)
Others (no-headache)	12/41 (29.2 %)	15/58 (25.9 %)	14/63 (22.2 %)
No symptom	12/41 (29.2 %)	27/58 (46.6 %)	40/63 (63.5 %)

Note: % indicates the incidence of symptom

**Table 2:** Cytokines level at 540 m and at 3860 m (hypoxia)

	TNF-α		IL-1β		IL-6 (pg/ml)		*N*
Symptoms	540 m	3860 m	540 m	3860 m	540 m	3860 m	74
No-AMS:							
A (no symptom)	15.09 ± 0.51 (3.22)	19.39 ± 1 (6.33)***	3.43 ± 0.63 (2.1)	5.07 ± 0.98 (3.24)***	7.51 ± 0.36 (2.28)	11.43 ± 0.8 (5.06)***	40
B (headache only)	17.57 ± 2.25 (5.52)	22.7 ± 2.83 (6.94)*	4.1 ± 0.81 (1.98)	7.23 ± 1.01 (2.48)	8.57 ± 0.98 (2.4)	18.27 ± 3.65 (8.94)*^,+^	6
C (headache + 1 symptom)	16.64 ± 3.24 (5.61)	20.31 ± 4.55 (7.88)	4.32 ± 0.37 (0.64)	10.22 ± 2.51 (4.35)^$^	7.85 ± 1.23 (2.13)	15.2 ± 2.59 (4.48)	3
D (other symptoms)	15.46 ± 1.18 (4.4)	20.96 ± 1.32 (4.92)***	4.52 ± 0.58 (2.16)	7.12 ± 0.55 (2.07)***^,@^	7.47 ± 0.57 (2.12)	13.32 ± 1.49 (5.57)**^,^^&^	14
AMS:							
E (headache + 2 symptoms)	15.23 ± 1.24 (4.11)	25.59 ± 2.84 (9.42)**^, #^	3.34 ± 0.46 (1.51)	7.65 ± 0.85 (2.83)***^,#^	8.14 ± 0.76 (2.53)	20.62 ± 2.5 (8.29)***^,###^	11

Values are presented means ± SE (SD)

*P < 0.05; **P < 0.01; ***P < 0.001 compared with cytokines level of same group at 540 m

^#^ *P* < 0.05; ^###^ *P* < 0.001 compared with A group (TNF, *P* = 0.013; IL-1, *P* = 0.02; IL-6, *p* = 7.6 × 10^−5^) at 3860 m

^+^ *P* < 0.017; compared with A group at 3860 m by *Bonferroni’s* multiple comparisons test after correction (IL-6, A vs B *P* = 0.008)

^$^ *P* < 0.017, trends by 0.017 < ^@^ *P* < 0.05 (IL-1, A vs C, *P* = 0.013; A vs D *P* = 0.03)

^&^ *P* < 0.05 compared with E group (D vs E, *P* = 0.02) at 3860 m

### Peripheral LPS-induced systemic inflammation promotes the development of brain edema in rats under hypoxia

**Fig. 2:**
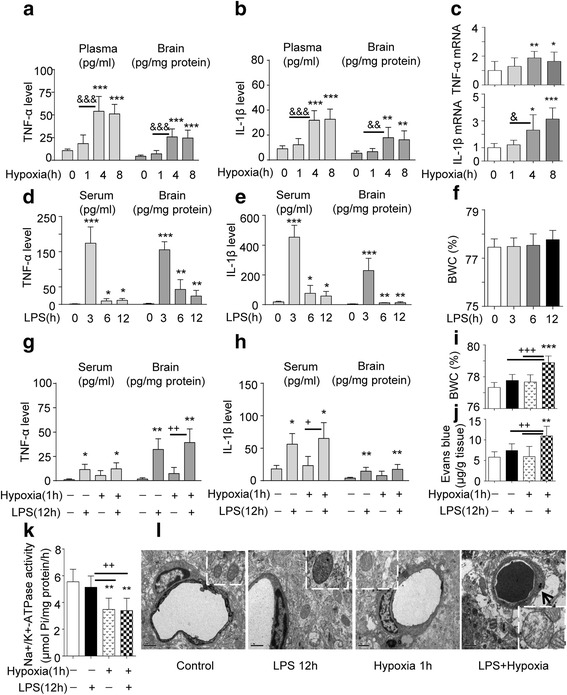
Systemic inflammation facilitates the onset of brain edema under transient hypoxia. **a**, **b** TNF-α and IL-1β level in serum and in brain cortex of rats were tested after hypoxia exposure (1–8 h) and increased after 4 h. **c** Hypoxia-increased TNF-α and IL-1β mRNA expression in brain cortex (*n* = 6). *P < 0.05; **P < 0.01; ***P < 0.001 compared with control. ^&^ *P* < 0.05; ^&^^&^ *P* < 0.01; ^&&&^ *P* < 0.001 (4 h hypoxia vs 1 h hypoxia). **d**, **e** TNF-α and IL-1β levels in serum and cortex of rats were measured by ELISA and increased, following the LPS treatments indicated (*n* = 6). *P < 0.05; **P < 0.01; ***P < 0.001 compared with control. **f** Brain water content (BWC) was determined by wet/dry weight ratio at the time points indicated after LPS treatment (*n* = 6–12). **g**, **h** TNF-α and IL-1β levels in serum and cortex of rats were measured by ELISA and increased, following LPS or hypoxia alone or combination of both treatments indicated (*n* = 6). *P < 0.05; **P < 0.01; compared with control. ^+^ *P* < 0.05; ^++^ *P* < 0.01, hypoxia + LPS vs hypoxia. **i** BWC was determined by wet/dry weight ratio after LPS, hypoxia or both (*n* = 6–12), ***P < 0.001 compared with control, ^+++^ *P* < 0.001, compared with LPS or hypoxia alone. **j** BBB permeability was determined by detecting the extravasations of Evans blue dye (*n* = 8). **P < 0.01 compared with control, ^++^ *P* < 0.001, compared with LPS or hypoxia alone. **k** Na^+^/K^+^-ATPase activity in brain cortex was measured following the indicated treatments (*n* = 6). **P < 0.01 compared with control, ^++^ *P* < 0.01, compared with hypoxia or hypoxia + LPS. All data are presented as mean ± SD. **l** Representative transmission electron micrographs of perivascular astrocytes and mitochondria. *Scale bar*, 1 μm, *arrow* indicates enlarged astrocyte foot processes

To investigate the potential role of systemic inflammation in the development of brain edema under hypoxia, four groups of rats were exposed to normoxia or hypoxia (altitude of 7000 m) in a hypobaric chamber: one normoxic group, one hypoxic group, one LPS group (ip, 4 mg/kg) under normoxia, and one LPS + hypoxia group. First, hypoxia exposure for 4 or 8 h markedly increased TNF-α and IL-1β levels in both plasma and brain cortex (Fig. 2a, b) and TNF-α mRNA and IL-1β mRNA expressions in brain cortex, compared with control or hypoxia for 1 h group (Fig. 2c). Second, a single injection of LPS to rats for 0, 3, 6, 12 h, induced systemic pro-inflammatory response under normoxia, showing significantly increased TNF-α and IL-1β levels in both plasma and brain cortex (Fig. 2d, e) compared with control but did not increase BWC (Fig. 2f). Third, rats were exposed to hypobaric hypoxia for 1 h, and this did not increase the cytokine levels or BWC but reduced Na^+^-K^+^-ATPase activity (Fig. 2g–i, k). However, rats pre-treated with LPS alone for 11 h followed by 1 h hypobaric hypoxia (7000 m) (LPS + hypoxia) showed markedly increased TNF-α and IL-1β levels in the plasma and brain cortex (Fig. 2g, h) and increased BWC (Fig. 2i) as well as Evans blue extravasations (Fig. 2j), compared with LPS injection alone, suggesting LPS + hypoxia induces BBB disruption and plays a role in induction of BWC (vasogenic edema). In addition, hypoxia exposure alone (altitude, 7000 m, 1 h) significantly decreased Na^+^/K^+^-ATPase activity in rat brain cortex (Fig. 2k) and caused water influx into brain cells because of hyperosmotic pressure in cells, revealed by further swelling of astrocytes. LPS + hypoxia simultaneously induced cytotoxic and vasogenic edema, which was supported by the transmission electron microphotograph (Fig. 2l), showing an enlarged perivascular space of astrocyte endfeet in rat cortex compared with single LPS or hypoxia treatment. Moreover, water overloaded rats by ip 20 % ddH_2_O showed increased BWC, which became more severe when challenged by LPS, indicating LPS played a role in induction of brain edema but required extracellular hyposmolarity (Additional file 1: Figure S1A). GFAP-labeled astrocytes presented thicker foot processes in LPS + hypoxia rats (Additional file 1: Figure S1B). LPS + hypoxia-induced swellings of perivascular astrocyte endfeet (Fig. 2l) were also attenuated by PDTC (Additional file 1: Figure S1B, last one). These results suggest that the increase of cerebral vascular permeability and brain edema is a result of a combination of LPS-increased pro-inflammatory cytokines and hypoxia-increased vessel permeability together with BBB disruption and hypoxia-reduced Na^+^/K^+^-ATPase activity.

### LPS increases expression of AQP4 and AQP4 mRNA in rat cortex and astrocytes and water permeability of astrocytes

**Fig. 3:**
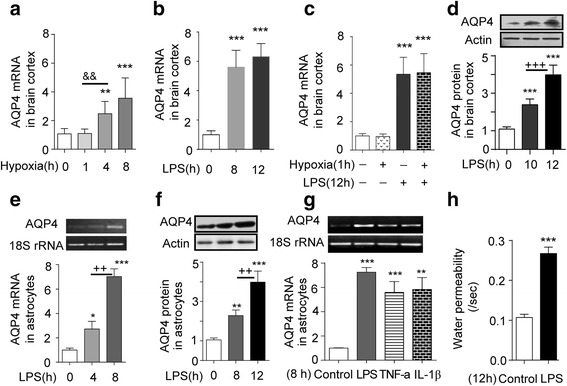
LPS and cytokines increase AQP4 mRNA and protein expression in the brain cortex and astrocytes, and LPS increases water permeability of astrocytes. **a** The expression of AQP4 mRNA in brain cortex tested by Q-PCR following hypoxia exposure for the time points indicated (*n* = 6). **P < 0.01, ***P < 0.001 compared with control; ^&^^&^ *P* < 0.01 (hypoxia 4 h vs hypoxia 1 h). **b** The expression of AQP4 mRNA in the brain cortex of rats determined by Q-PCR following injection of LPS for the time points indicated (*n* = 6). ***P < 0.001 compared with control. **c** Hypoxia + LPS and LPS increased AQP4 mRNA expression in brain cortex (by Q-PCR), ***P < 0.001 compared with control. **d** AQP4 protein expression in brain cortex was detected by western blot after LPS treatment at the time points indicated (*n* = 6). ***P < 0.001compared with control, ^+++^ *P* < 0.001 vs LPS 10 h. **e** AQP4 mRNA expression and **f** AQP4 protein expression in astrocytes were detected by RT-PCR and western blot after LPS challenge (*n* = 3). *P < 0.05; **P < 0.01; ***P < 0.001, compared with control; ^++^ *P* < 0.01, compared with 8 h. **g** AQP4 mRNA expression in astrocytes was determined by RT-PCR after LPS, TNF-α, or IL-1β treatment for 8 h (*n* = 3). **P < 0.01, ***P < 0.001, compared with control. **h** Water permeability of cells increased by LPS challenge (*n* = 100). ***P < 0.001, compared with control. All the data are presented as mean ± SD

Considering the essential role of AQP4 in the formation of cytotoxic edema, we measured its expression in brain cortex and astrocytes and found that hypoxia (7000 m elevation, 4 or 8 h) increased AQP4 mRNA in cortex (Fig. 3a), and LPS (4 mg/kg, 8 or 12 h) also markedly increased its expression (Fig. 3b). Furthermore, LPS 12 h + hypoxia 1 h significantly increased AQP4 mRNA in cortex, compared with control, but the increase was no different to the increase caused by LPS alone. Hypoxia 1 h alone did not cause any significant effect compared to control (Fig. 3c). AQP4 protein and mRNA were time-dependently enhanced in brain cortex and astrocytes (Fig. 3d–f) after LPS treatment. In addition, AQP4 mRNA increased after TNF-α and IL-1β challenge for 8 h (Fig. 3g). Moreover, LPS also increased water permeability in astrocytes (Fig. 3h). To demonstrate that the highly expressed AQP4 was associated with enhanced water permeability of astrocytes, RFP-tagged AQP4 was transfected into HeLa cells and the membrane permeability was measured after hypo-osmotic challenge. This showed that the cell membrane permeability was positively correlated with AQP4 expression (Additional file 1: Figure S2A). Inhibitors of protein synthesis (CHX) and transcription (ActD) abolished the LPS-increased membrane permeability (Additional file 1: Figure S2B). The increase in BWC following LPS + hypoxia was also prevented by a PDTC or MG132 (Additional file 1: Figure S2C). These data suggest that stimulating AQP4 expression by pro-inflammatory factors increases water permeability in astrocytes.

### Inflammatory factors-induced increase of AQP4 expression through NF-κB and MAPK pathway

**Fig. 4:**
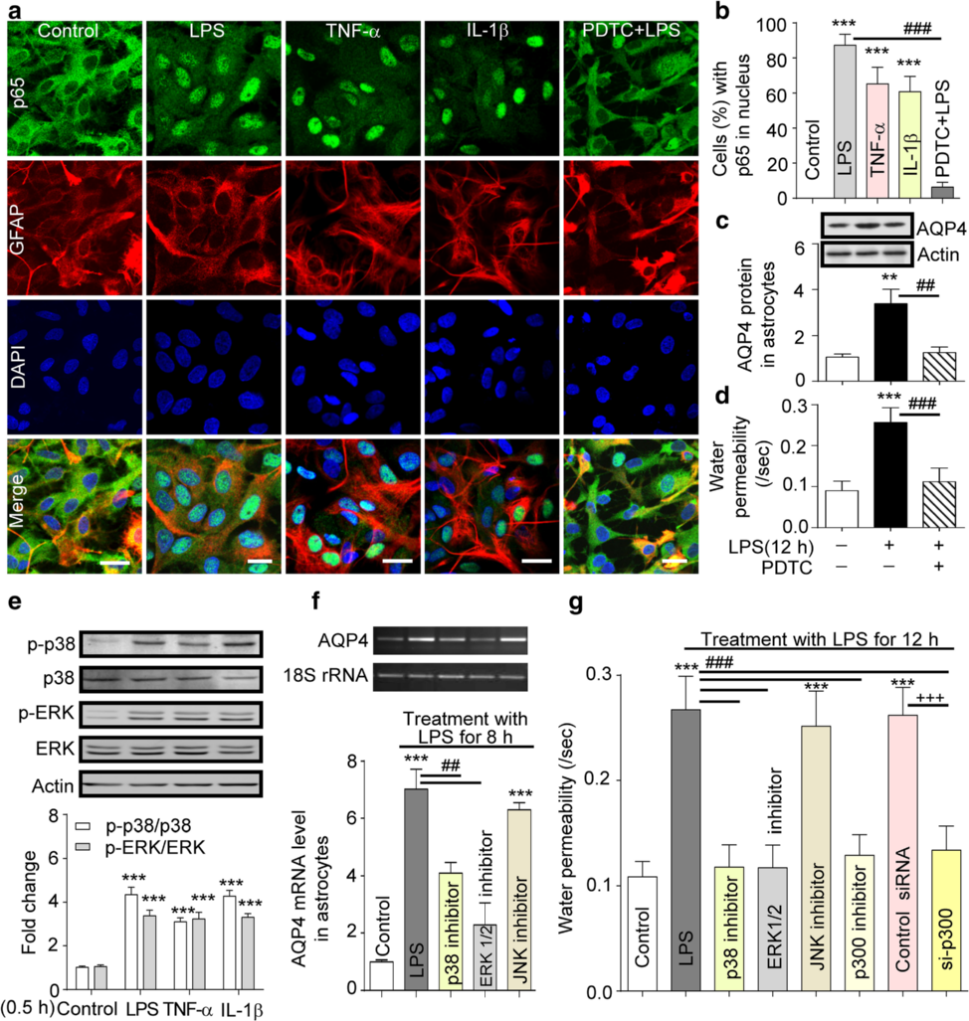
Inflammation induced increased AQP4 expression via the NF-κB and MAPKs pathway. **a** Astrocytes pre-treated with or without PDTC were treated with LPS, TNF-α, or IL-1β for 2 h, followed by staining with specific p65 antibody (*n* = 3). **b** p65 translocation into cell nuclei (%) after treatment with LPS, TNF-α, or IL-1β for 2 h. **c** AQP4 protein levels in astrocytes treated with LPS for 12 h after pre-treatment with or without PDTC (*n* = 3). **d** Water permeability of cells after LPS or LPS + PDTC treatment (*n* = 100). **P < 0.01; ***P < 0.001 compared with control; ^##^ *P* < 0.01;^###^ *P* < 0.001 compared with LPS 12 h. **e** Phosphorylated levels of ERKs and p38 in astrocytes treated with LPS, TNF-α, or IL-1β for 0.5 h were increased (*n* = 3). ***P < 0.001, compared with control. **f** AQP4 mRNA changes measured by RT-PCR in astrocytes treated with LPS for 8 h after pre-treatment with p38 inhibitor, ERK1/2 inhibitor, JNK inhibitor (*n* = 3). ***P < 0.001 compared with control; ^##^ *P* < 0.01 compared with LPS treatment. **g** Water permeability changes of astrocytes pre-treated with p38 inhibitor, ERK inhibitor, JNK inhibitor, p300 inhibitor, or transfected with p300 siRNA then exposure to LPS for 12 h (*n* = 100). ***P < 0.001 compared with control; ^###^ *P* < 0.001 compared with LPS 12 h. All the data are presented as mean ± SD

A clear translocation of p65 from cytoplasm to the nucleus was found in LPS, TNF-α, or IL-1β treated astrocytes compared with untreated astrocytes. PDTC pre-treatment prevented p65 translocation (Fig. 4a, b), inhibited AQP4 protein expression and blocked the increase of water permeability in the astrocytes (Fig. 4c, d), showing NF-κB involvement. We further demonstrated that LPS, TNF-α, or IL-1β all induced the phosphorylation of cellular signal regulated protein kinase (ERK)1/2, p38, and p300 activity (acetylation H3 and H4) (Fig. 4e and Additional file 1: Figure S3A). In addition, the inhibitors of p38 (SB203580), ERK1/2 (U0126), p300 (Curcumin, Cur), and si-p300 (Fig. 4f, Additional file 1: Figure S3B-D) blocked LPS-stimulated increase of AQP4 mRNA. Inhibitors of JNK (SP600125) did not change AQP mRNA and membrane permeability. The inhibitors of ERKs, p38, and p300 could also reduce the increase of astrocyte membrane permeability caused by LPS 12 h (Fig. 4g). Therefore, MAPKs (ERKs and p38) and p300 play important roles in the increase of AQP4 and astrocyte membrane permeability induced by inflammation.

### CRH stimulates microglia and triggers immune-inflammatory response under hypoxia

**Fig. 5:**
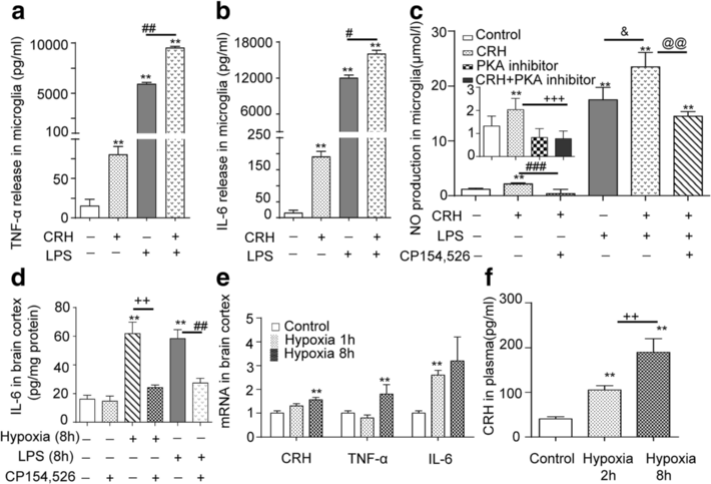
CRH stimulates microglia and triggers an immune-inflammatory response. **a**, **b** Increase in TNF-α and IL-6 release in cultured primary cortical microglia induced by CRH or LPS or CRH+LPS challenge for 24 h. **P < 0.01, compared with control, ^#^ *P* < 0.05; ^##^ *P* < 0.01, LPS + CRH vs LPS. **c** Increase in NO production in cultured primary cortical microglia challenged by CRH, LPS or CRH+LPS. **P < 0.01, compared with control; &*P* < 0.01, compared between LPS + CRH and LPS; ^@@^ *P* < 0.01 compared between CRH + LPS and CRH + LPS + CP154,526; ^###^ *P* < 0.01, compared between CRH and CRH + CP154,526. CRH stimulated increased NO production, was blocked by PKA inhibitor (*inset*). ***P < 0.001 compared with control, ^+++^ *P* < 0.001 compared between CRH and CRH + PKA inhibitor. All the data are presented as mean ± SD. **d** Hypoxia and LPS increased IL-6 protein levels in brain cortex. Increased IL-6 protein in rat brain cortex following either hypoxia or LPS (8 h) was blocked by CRHR1 antagonist, *n* = 6, **P < 0.01 compared with control, ^++^ *P* < 0.01 compared between hypoxia and hypoxia + CP154,526, ^##^ *P* < 0.01 compared between LPS and LPS + CP154,526, the data are presented as mean ± SD. **e** Hypoxia stimulated expression of mRNAs of CRH, TNF-α, and IL-6 in brain cortex (*n* = 6) **P < 0.01, compared with control, the data are presented as mean ± SD. **f** Hypoxia-increased CRH release in circulation **P < 0.01, compared with control; ^++^ *P* < 0.01, compared between hypoxia 2 and 8 h, the data are presented mean ± SD

To determine the effects of CRH on cerebral microglia, the release of TNF-α, IL-6, and NO from cultured primary microglia was measured. We found that CRH challenge for 24 h induced TNF-α, IL-6, and NO release, and CRH + LPS induced further increase of these cytokines compared with CRH and LPS alone (Fig. 5a, b). The increase in NO release elicited by CRH was blocked by CRHR1 antagonist (CP 154,526) (Fig. 5c) and by PKA pathway inhibitor (insert in Fig. 5c). CRH + LPS-induced release of NO was reduced by CRHR1 antagonist CP154,526 (Fig. 5c). Increased IL-6 protein in brain cortex after hypoxia or LPS 8 h was blocked by CRHR1 antagonist (CP154,526) (Fig. 5d). In vivo, hypoxia (1or 8 h at altitude of 7000 m) also significantly increased CRH, TNF-α, and IL-6 mRNA expression in rat brain cortex (Fig. 5e) and this hypoxia-increased plasma CRH level in a time-dependent manner (Fig. 5f). These data evidence that both CRH and LPS have a stimulating effect on cytokine release in cerebral microglia.

## Discussion

HACE is a severe form of AMS; untreated severe AMS or treated non-appropriately may progress to HACE. Increasing numbers of papers suggest that an inflammatory response is associated with AMS and HACE, but the exact mechanisms are less explored and the inflammatory view remains speculative. This study attempted to provide credible evidence for a pathogenic role of inflammatory mediators in AMS and more particularly in HACE. The experiments showed that high-altitude hypoxia induces an increase of circulating cytokines TNF-α, IL-1β, and IL-6 in humans. We also found that the levels of TNF-α and IL-1β were increased in rat plasma and brain cortex (Figs. 1 and 2), and their gene expression was increased in cortex (Fig. 2) and increased in a time-dependent manner in whole blood by simulated altitude hypoxia (data not shown), suggesting an enriched way for increasing their plasma levels. More importantly, the plasma stress peptide, CRH levels, increased significantly in both human and rats. This was accompanied by positively associated upregulated pro-inflammatory factors and was blocked by CRHR1 antagonist. Significantly, the study showed, for the first time, that the magnitude of the pro-inflammatory factors of TNF-α, IL-1β, and IL-6 in humans was positively correlated with the development of the symptoms of AMS (Fig. 1c). In accord with these results, other studies have suggested that hypoxia-elicited neurohormonal changes ultimately lead to increased cerebral blood flow, enhanced permeability of the BBB, and cerebral edema [1, 2, 8, 26–28]. Furthermore, it is known that hypoxia increases levels of circulating pro-inflammatory cytokines and ROS markers, with vascular leakage, suggesting that these changes may be the causes of cerebral edema observed in volunteers at high altitude [29–33]. The new findings of the present study allow us to propose a novel pathological theoretical perspective for AMS and even HACE, which is that hypoxia-activated CRH and its CRHR1 signaling play a key role in induction of a pro-inflammatory response and AMS. Others have also linked cytokine activation with effects on brain function. For example, in a recent review, it was suggested that hypoxia of high altitude and its association with increased TNFα, IL-1β, and IL-6 might explain sleep disturbances, but a link with AMS and HACE [34] was not implied. We are also aware there is report that AMS susceptibility seems not related to an exaggerated inflammatory response [33]. This may associate with the group size (20 vs 74 in the present study), age (25–31 years old vs 18–23 years old), gender (men + women vs men), the duration (4.9 h vs 3 days), and altitude (4875 vs 3860 m).

It is clear from the present study that plasma cytokine levels on human exposure to high altitude were increased and positively correlated with LLS score (Fig. 1c, 3860 m). The level of inflammatory markers (plasma TNF-α, IL-1β, IL-6) showed a significant separation between the no-symptoms group and AMS group (Table 2). Therefore, we consider that significant high plasma level of cytokines TNF-α, IL-1β, and IL-6 could be valuable for evaluating AMS compared to no-AMS in humans exposed to high altitude. In addition, since it is known that the development of AMS largely associates with the elevation, speed of ascent, and the duration at the altitude, these would make the changes in cytokines flexible.

AMS and HACE are part of a spectrum of altitude illness. AMS can develop into cerebral edema when treated improperly or if a person suffers more severe hypoxia [1–5]. Both forms of high-altitude illness are exacerbated when combined with infection-elicited inflammation. Therefore, we studied the impact of hypoxia with or without infection-elicited immune-inflammation on the onset and extent of cerebral edema during transient or prolonged hypoxia. For this, we used the rat as a model because ethically we could not take human volunteers to an altitude at which there might be a greater chance of inducing HACE without good medical and emergency evacuation facilities as well as a plentiful supply of oxygen. The rat also gave us the opportunity to look at the effects of exposure at different altitudes and for different durations using our well-designed automatically controlled hypobaric chamber with which we have much experience. The rat model enabled us to examine the interaction of signaling pathways involved in hypobaric hypoxia and infection induced inflammation. For the present purpose, we established a rat inflammation model using ip injection of LPS (4 mg/kg, BW) to mimic an infection and found that LPS significantly increased TNF-α and IL-1β levels in the plasma and brain cortex. The experiment also revealed that LPS or pro-inflammatory factors increase AQP4 and AQP4 mRNA through TLR4 signaling via MAPKs and NF-κB in cortex and astrocytes, shown by these pathways being blocked by selective antagonists. Associated with this signaling there is increased water permeability of astrocytes via activated NF-κB when charged by hypotonic solution. It would therefore seem that inflammation at some level provokes the onset of cerebral edema. However, LPS alone, that is without hypoxia, did not induce brain edema, which clearly indicates that both stimuli are needed for brain swelling. This may of course depend on the dosage and time course, the method of treatment with LPS or, perhaps, the animal strain used. For example, in other studies, 250 μg (0.5 ml) or 4 mg/kg of LPS given ip did not induce cerebral edema in adult rats [20–22, 35], whereas microinjection of LPS directly into the substantia nigra of rat brain (2 μl, 1 mg/ml) or injection of LPS in mice (ip, 0.15 mg/mouse) induced brain edema [36]. In humans, inflammation can induce cerebral edema in stroke or trauma [37, 38], but importantly, these are accompanied by local hypoxia. In accord, cerebral edema is observed with other ischaemic hypoxic disease. Hence, the balance of evidence suggests that inflammation has an essential pathogenic significance in brain edema.

Using the rat model in the present study has revealed some of the factors and mechanisms that are a likely cause of the cerebral edema, associated with hypoxia and inflammation. Clearly duration of stress is important. Thus, hypoxia (7000 m, ~8.2 % O_2_) for 1 h reduced Na^+^-K^+^-ATPase activity (Fig. 2) but did not induce a pro-inflammatory response; however, hypoxia for 4 and 8 h induced inflammation and cerebral edema (Fig. 2). Moreover, a more severe challenge, LPS + hypoxia (LPS for 11 h prior to 1 h hypoxia) resulted in cerebral edema. This appeared to be due to LPS-triggered cytotoxic astrocyte swelling caused by activating AQP4 and by hypoxia-reduced Na^+^-K^+^-ATPase. This allowed increased water influx into cells. Hence, LPS, combined with hypoxia-induced BBB vasculature leakage, enhances edema. In accord with this in humans, hypoxia has been shown to increase cerebral blood flow in the human brain as measured by single photon emission computed tomography [39]. In rats, inflammation causes astrocyte swelling with disruption of the BBB [40–42]. Thus, there is a clear parallel between the experimental data in rats and that seen in humans. Importantly, from the rat experiments, we can gain an idea of the cellular mechanisms that may lead to HACE. For example, prolonged hypoxia increases the cytokine (Fig. 2g–i) and CRH levels in plasma and enhances CRH, IL-6, and TNF-α mRNA expression in brain (Fig. 5e, f). In addition, hypoxia or LPS can upregulate IL-6 protein in the brain cortex (Fig. 5d). Also CRH or LPS, particularly CRH + LPS, stimulate TNF-α, IL-6, and NO release in cultured primary microglia via CRH-activated CRHR1 signaling PKA. These data suggest that both an infection- and hypoxia-elicited inflammatory response can summate to worsen cerebral edema through interaction between astrocytes and microglia. This depends on TLR4-initiated MAPKs and NF-κB and CRHR1-initiated cAMP/PKA signaling to activate AQP4 and eventually induce cytotoxic and vasogenic edema.

**Fig. 6:**
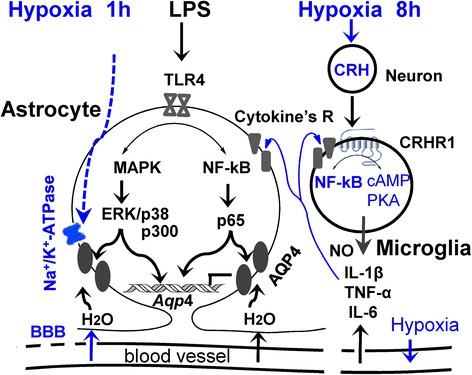
Integrated results of this study to diagrammatically show how LPS-induced systemic inflammation facilitates the onset of cerebral edema under short-term hypoxia in rats by (1) LPS-induced pro-inflammatory pathway which activates AQP4 and contributes to water permeability of astrocytes via TLR4 signaling MAPKs and NF-κB, (2) short-term hypobaric hypoxia which reduced Na^+^-K^+^-ATPase activity resulting in water inflow in astrocytes, (3) LPS + hypoxia-induced leakage of BBB resulting in vasogenic edema. All together, these factors contribute to brain edema. Under prolonged hypoxia, systemic inflammation may worsen the cerebral edema by (1) LPS plus hypoxia-induced astrocyte cytotoxic swelling; (2) LPS plus hypoxia-activated cortical microglia release of cytokines, which further stimulate astrocyte swelling; and (3) hypoxia further reducing Na^+^-K^+^-ATPase activity, and LPS + hypoxia triggering leakage of BBB. This mechanism in rats may describe the onset and deterioration of HACE in human

We have recently reported that in rats, hypoxia-induced overactivation of CRHR1 and AQP4 produces cerebral edema tested by magnetic resonance imaging (MRI). This action is mediated by hypoxia activating local CRH neurons in cortex through CRHR1 initiating cAMP/PKA and Ca^2+^/PKCε signaling which triggers AQP4 phosphorylation and gene expression in astrocytes to contribute to brain edema [8]. The protein kinase G (PKG) pathway is not directly involved in activation of AQP4 in astrocytes as hypoxia-induced HIF-1α expression does not cause iNOS expression and NO release in astrocytes [8]. However, we showed in present study that NO is released and supplied by hypoxia-activated microglia (Fig. 5), which implies that the PKG pathway also contributes to astrocyte swelling. These data are summarized in Fig. 6f and showed how an interplay between astrocyte and microglia through TLR4 and CRHR1 signaling pathways work during infection and hypoxia to cause cerebral edema.

## Conclusions

The present study shows that in humans and rats, hypobaric hypoxia causes an inflammatory response with cytokine release, which positively correlates to severity of AMS syndrome in humans and with activation of CRHR1 in central microglia and peripheral tissues in rats. The experiments have also clarified the mechanisms that lead to cerebral edema associated with hypobaric hypoxia and provide for the first time clear evidence for a facilitatory role of inflammation. These results suggest that the CRH receptor CRHR1 might be a promising target for prevention and therapy of AMS and HACE and enable development of novel therapeutic interventions with wider clinical potential.

## Additional files

Additional file 1: Figures S1–S3. Figure S1. (A) Brain water content was measured following injection of rats with LPS (ip) for 12 h, H_2_O-injection for 0.5 h or both (*n* = 6). ****p* < 0.001 compared with control; ^###^ *P* < 0.001 compared between 20 % ddH_2_O and 20 % ddH_2_O + LPS treatment. (B) Immunohistochemical fluorescence of GFAP in the cortex of rats treated as indicated. Scale bar, 50 μm. Representative transmission electron micrographs of perivascular astrocytes and mitochondria. Scale bar, 1 μm (last one). Figure S2. (A) Fluorescence intensity of RFP-AQP4 and water permeability were measured in transfected RFP-AQP4 HeLa cells (*n* = 60). (B) Astrocytes were pre-treated with CHX or ActD or untreated. The water permeability of astrocytes was determined after 12 h LPS stimulation (*n* = 100). ****P* < 0.001 compared with control; ^###^ *P* < 0.001 compared with LPS 12 h. (C) Brain water content was determined by dry-wet weight method at the indicated time points and under the indicated conditions (*n* = 6–12). ****p* < 0.001 compared with control; ^###^ *P* < 0.001 compared with LPS + hypoxia, all data are presented as mean ±SD. Figure S3. Inflammation induced AQP4 high expression through MAPK pathway. (A) Astrocytes were treated with LPS as indicated time, and the phosphorylation levels of ERKs and p38 were tested by western blot using anti-phospho-ERK or anti-phospho-p38 antibodies (*n* = 3). **P* < 0.05; ***P* < 0.01;****P* < 0.001 compared with control. (B) Acetylation levels of H3 and H4 in astrocytes were determined by western blot as indicated time after LPS treatment (*n* = 3). ****P* < 0.001 compared with control, Student’s *t* test. (C,D) Increased-AQP mRNA by LPS in astrocytes were be blocked by Cur, si-p300. ****P* < 0.001 compared with control, ^###^ *P* < 0.001 compared with LPS or si control, all data are presented as mean ±SD. (DOC 1687 kb)

## Abbreviations

AMS: acute mountain sickness
AQP4: aquaporin-4
BBB: blood-brain barrier
BWC: brain water content
CRH: corticotrophin-releasing hormone
CRHR1: corticotrophin-releasing hormone receptor type 1
HACE: high-altitude cerebral edema
LLS: Lake Louise Score
PDTC: pyrrolidine dithiocarbamate
PKG: protein kinase G
