# Supplementary material for: Systemic pro-inflammatory response facilitates the development of cerebral edema during short hypoxia
Source: J Neuroinflammation. 2016 Mar 11;13:63. doi: 10.1186/s12974-016-0528-4 (PMC4788817; doi:10.1186/s12974-016-0528-4)
Supplement: Additional file 1: Figures S1–S3. — Figure S1. (A) Brain water content was measured following injection of rats with LPS (ip) for 12 h, H2O-injection for 0.5 h or both (n = 6). ***p < 0.001 compared with control; ### P < 0.001 compared between 20 % ddH2O and 20 % ddH2O + LPS treatment. (B) Immunohistochemical fluorescence of GFAP in the cortex of rats treated as indicated. Scale bar, 50 μm. Representative transmission electron micrographs of perivascular astrocytes and mitochondria. Scale bar, 1 μm (last one). Figure S2. (A) Fluorescence intensity of RFP-AQP4 and water permeability were measured in transfected RFP-AQP4 HeLa cells (n = 60). (B) Astrocytes were pre-treated with CHX or ActD or untreated. The water permeability of astrocytes was determined after 12 h LPS stimulation (n = 100). ***P < 0.001 compared with control; ### P < 0.001 compared with LPS 12 h. (C) Brain water content was determined by dry-wet weight method at the indicated time points and under the indicated conditions (n = 6–12). ***p < 0.001 compared with control; ### P < 0.001 compared with LPS + hypoxia, all data are presented as mean ±SD. Figure S3. Inflammation induced AQP4 high expression through MAPK pathway. (A) Astrocytes were treated with LPS as indicated time, and the phosphorylation levels of ERKs and p38 were tested by western blot using anti-phospho-ERK or anti-phospho-p38 antibodies (n = 3). *P < 0.05; **P < 0.01;***P < 0.001 compared with control. (B) Acetylation levels of H3 and H4 in astrocytes were determined by western blot as indicated time after LPS treatment (n = 3). ***P < 0.001 compared with control, Student’s t test. (C,D) Increased-AQP mRNA by LPS in astrocytes were be blocked by Cur, si-p300. ***P < 0.001 compared with control, ### P < 0.001 compared with LPS or si control, all data are presented as mean ±SD. (DOC 1687 kb) [file 12974_2016_528_MOESM1_ESM.doc]

**Supplementary Material**

**Methods**

Animal Hypoxia Treatment

The MG132(NF-κB inhibitor)+LPS+Hypoxia group (n = 10) was pre-treated with MG132 for 30 min then LPS treatment for 11 h, followed by hypoxia for 1 h. 2. The MG132+Hypoxia group (n = 6) was injected with MG132 for 30 min then exposed to hypoxia for 1 h.

Reagents, Antibodies and Plasmid

The gene transcription inhibitor actinomycin D (ActD), the protein synthesis inhibitor cycloheximide (CHX), MG132 were from Sigma (USA). For CHX (10 μg/ml) or ActD (20 μg/ml) the cells were pre-treated for 6 h. MG132 (1 μM), SB (10 μM), U0 (10 μM) or SP (1 μM) the cells were pre-treated for 0.5 h. For curcumin (20 μM) the cells were pre-treated for 24 h.

**Figure S1-S3 and Legends**


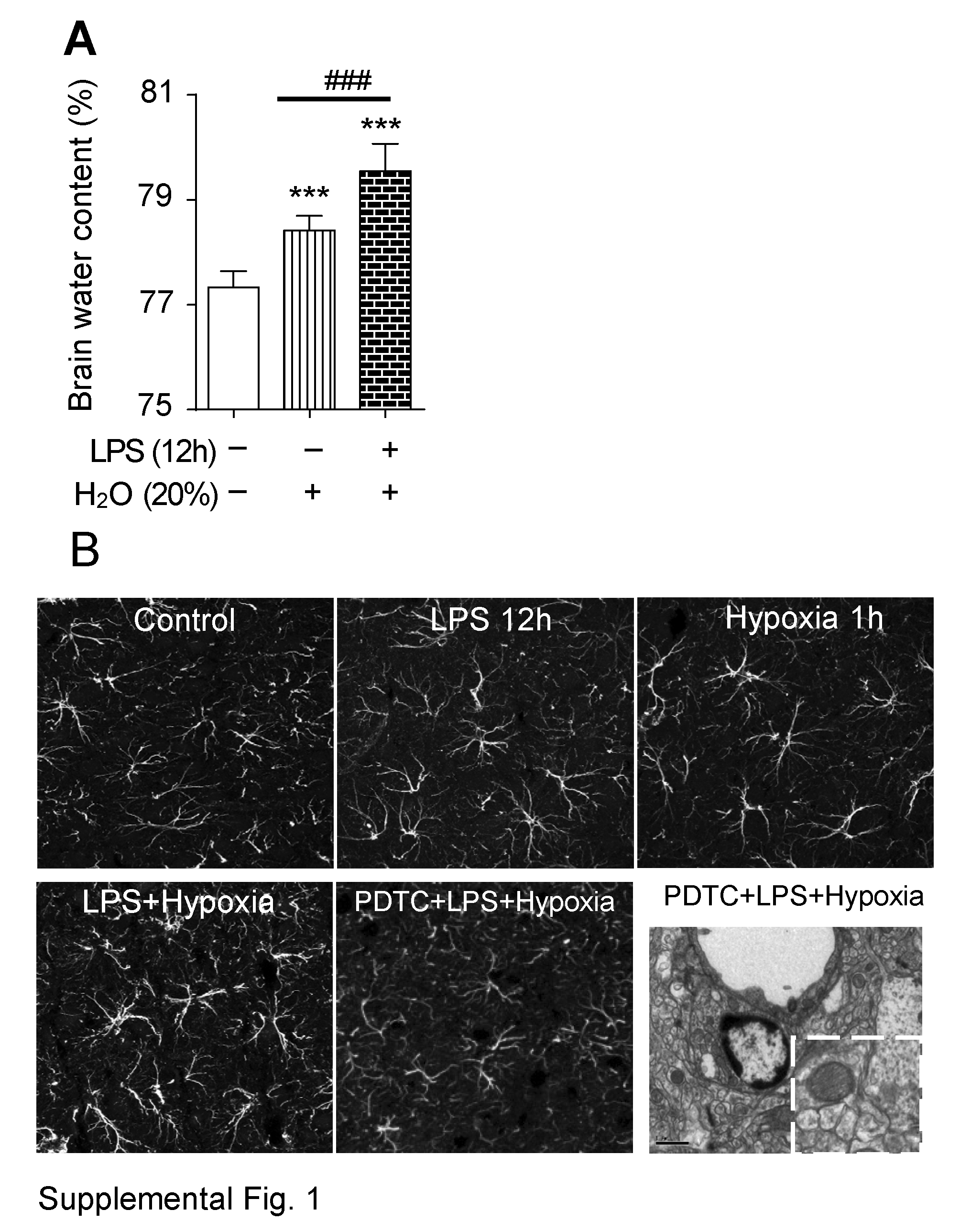


**Figure S1.** (**A**) Brain water content was measured following injection of rats with LPS (i.p.) for 12 h, H_2_O-injection for 0.5 h or both (n=6). ^***^*P* < 0.001 compared with control; ^###^*P* < 0.001compared between 20% ddH_2_O and 20% ddH_2_O + LPS treatment. (**B**) Immunohistochemical fluorescence of GFAP in the cortex of rats treated as indicated. Scale bar, 50 μm. Representative transmission electron micrographs of perivascular astrocytes and mitochondria. Scale bar, 1 μm (last one).


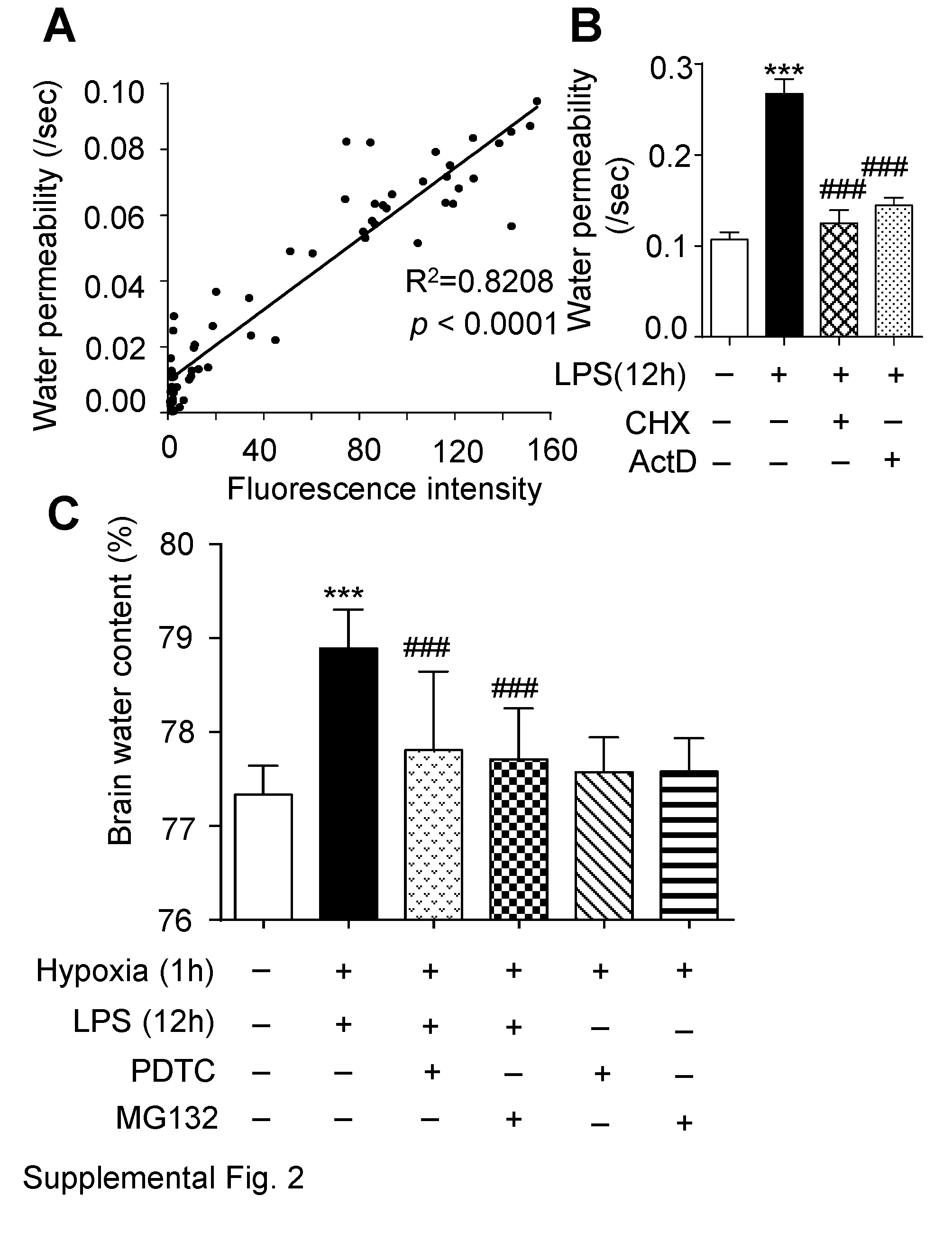


**Figure S2.** (**A**) Fluorescence intensity of RFP-AQP4 and water permeability were measured in transfected RFP-AQP4 HeLa cells (n = 60). (**B**) Astrocytes were pre-treated with CHX or ActD or untreated. The water permeability of astrocytes was determined after 12 h LPS stimulation (n=100). ^***^*P* < 0.001 compared with control; ^###^*P* < 0.001 compared with LPS 12 h. (**C**)Brain water content was determined by dry-wet weight method at the indicated time points and under the indicated conditions (n=6-12). *^***^P* < 0.001 compared with control; ^###^*P* < 0.001 compared with LPS+Hypoxia, all data are presented as mean ±SD.


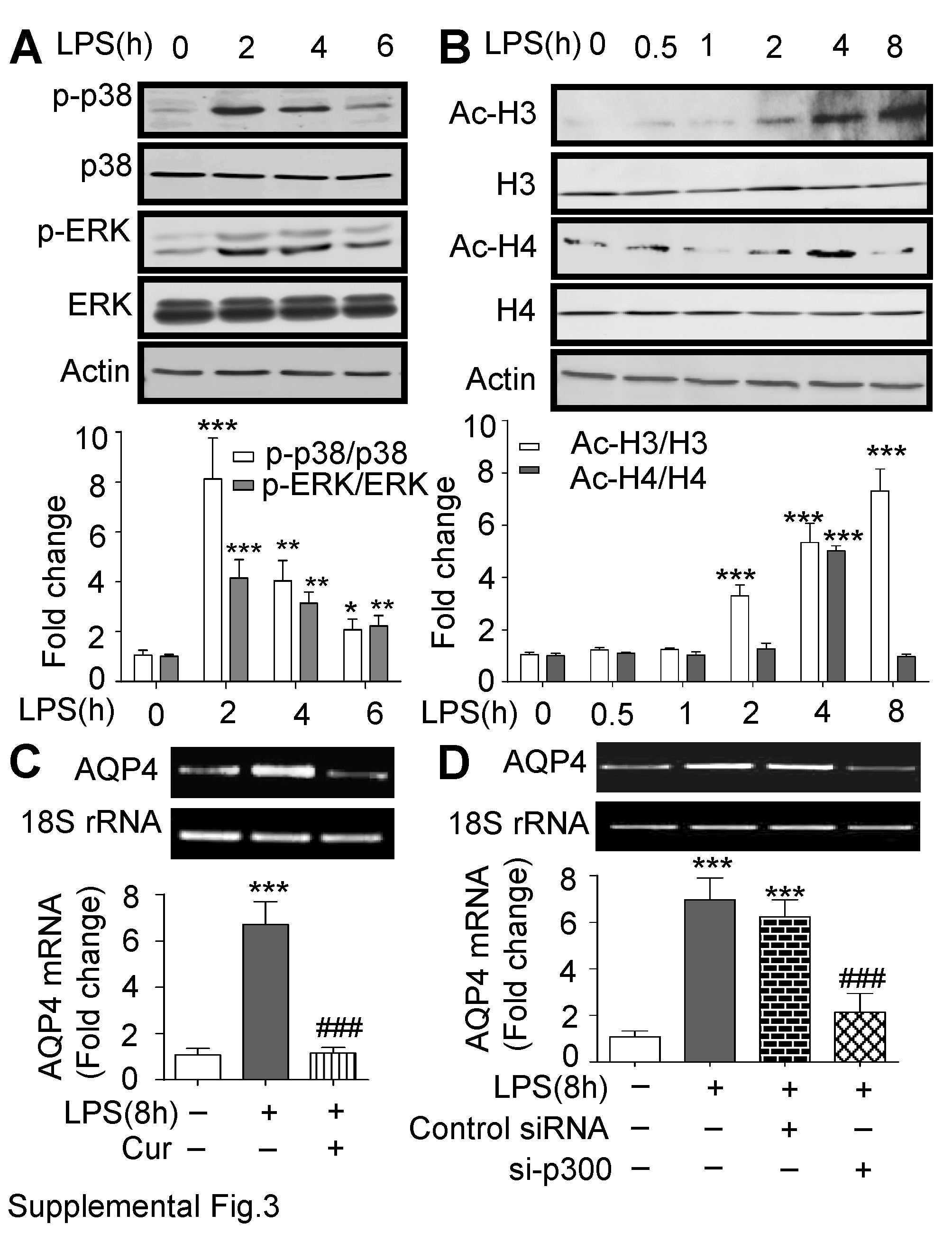


**Figure S3.** Inflammation induced AQP4 high expression through MAPK pathway. (**A**) Astrocytes were treated with LPS as indicated time, and the phosphorylation levels of ERKs and p38 were tested by western blot using anti-phospho-ERK or anti-phospho-p38 antibodies (n=3). ^*^*p* < 0.05; ^**^*p* < 0.01;^***^*p* < 0.001 compared with control. (**B**) Acetylation levels of H3 and H4 in astrocytes were determined by western blot as indicated time after LPS treatment (n=3). ^***^*p* < 0.001 compared with control, Student’s *t* test. (**C,D**) Increased-AQP mRNA by LPS in astrocytes were be blocked by Cur, si-p300. ^***^*p* < 0.001 compared with control, ^###^*p* < 0.001 compared with LPS or si control, all data are presented as mean ±SD.
